# 4-Cyano­pyridinium chloride

**DOI:** 10.1107/S1600536812018648

**Published:** 2012-05-05

**Authors:** Wen-Ni Zheng

**Affiliations:** a College of Chemistry and Chemical Engineering, Southeast University, Nanjing 210096, People’s Republic of China

## Abstract

In the crystal structure of the title salt, C_6_H_5_N_2_^+^·Cl^−^, the pyridinium cation links to the Cl^−^ anion *via* an N—H⋯Cl hydrogen bond. Weak C—H⋯Cl inter­actions also occur.

## Related literature

For the structures and properties of related compounds, see: Chen *et al.* (2000 ▶); Dai & Chen (2011 ▶); Xu *et al.* (2011 ▶); Liu *et al.* (1999 ▶); Zhao *et al.* (2003 ▶); Zheng (2011 ▶).
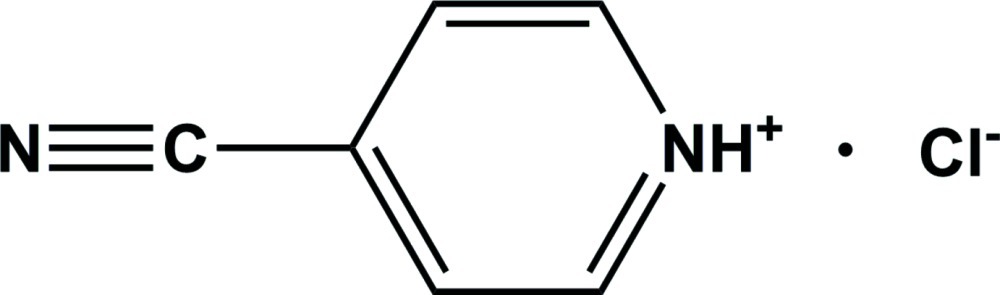


## Experimental

### 

#### Crystal data

C_6_H_5_N_2_^+^·Cl^−^
*M* *_r_* = 140.57
Triclinic, 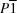

*a* = 6.6166 (2) Å
*b* = 7.6552 (3) Å
*c* = 8.3495 (5) Å
α = 63.957 (5)°
β = 69.830 (2)°
γ = 74.367 (4)°
*V* = 353.16 (3) Å^3^
*Z* = 2
Mo *K*α radiation
μ = 0.45 mm^−1^
*T* = 123 K
0.10 × 0.05 × 0.05 mm

#### Data collection

Rigaku Mercury2 diffractometer
Absorption correction: multi-scan (*CrystalClear*; Rigaku, 2005 ▶) *T*_min_ = 0.910, *T*_max_ = 1.000
3077 measured reflections
1231 independent reflections
1078 reflections with *I* > 2σ(*I*)
*R*_int_ = 0.042

#### Refinement

*R*[*F* ^2^ > 2σ(*F* ^2^)] = 0.086
*wR*(*F* ^2^) = 0.234
*S* = 1.32
1231 reflections
82 parameters
1 restraint
H-atom parameters constrained
Δρ_max_ = 0.97 e Å^−3^
Δρ_min_ = −0.62 e Å^−3^

### 

Data collection: *CrystalClear* (Rigaku, 2005 ▶); cell refinement: *CrystalClear*; data reduction: *CrystalClear*; program(s) used to solve structure: *SHELXTL* (Sheldrick, 2008 ▶); program(s) used to refine structure: *SHELXTL*; molecular graphics: *SHELXTL*; software used to prepare material for publication: *SHELXTL*.

## Supplementary Material

Crystal structure: contains datablock(s) I, global. DOI: 10.1107/S1600536812018648/xu5524sup1.cif

Structure factors: contains datablock(s) I. DOI: 10.1107/S1600536812018648/xu5524Isup2.hkl

Supplementary material file. DOI: 10.1107/S1600536812018648/xu5524Isup3.cml

Additional supplementary materials:  crystallographic information; 3D view; checkCIF report

## Figures and Tables

**Table 1:** Hydrogen-bond geometry (Å, °)

*D*—H⋯*A*	*D*—H	H⋯*A*	*D*⋯*A*	*D*—H⋯*A*
N1—H1⋯Cl1^i^	0.90	2.14	3.033 (5)	174
C4—H4*A*⋯Cl1	0.95	2.71	3.566 (5)	151
C5—H5*A*⋯Cl1^ii^	0.95	2.65	3.566 (6)	161

Symmetry codes: (i) 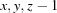; (ii) 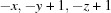.

**Fig. 1.**
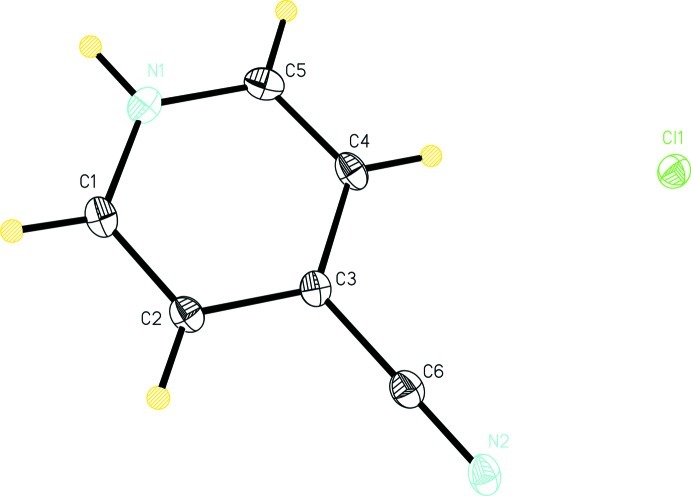
A view of the asymmetric unit with the atomic numbering scheme. The displacement ellipsoids were drawn at the 30% probability level.

## supplementary crystallographic information

### Comment

Simple organic salts containing strong intrermolecular H-bonds have attracted an attention as materials which display ferroelectric-paraelectric phase transitions (Chen *et al.*, 2000; Liu *et al.*, 1999; Zhao *et al.*, 2003). With the purpose of obtaining phase transition crystals of organic salts, various organic molecules have been studied and a series of new materials have been elaborated (Dai & Chen, 2011; Xu *et al.*, 2011; Zheng, 2011). Herewith we present the synthesis and crystal structure of the title compound.

In the title compound (Fig. 1), the bond lengths and angles have normal values. The asymmetric unit was composed of one 4-cyanopyridinium cation and one Cl^-^ anion. The protonated N atom was involved in strong intramolecular N—H···Cl hydrogen bonds with the N···Cl distance of 3.033 (5)Å. The weak intermolecular C4—H4A···Cl1 and C5—H45···Cl1 interactions were presented in the crystal structure with C5···Cl1 = 3.566 (5)Å and C5···Cl1 = 3.566 (6)Å, respectively. The crystal packing is further stabilized by aromatic π···π interactions between the pyridine rings of the neighbouring 4-cyanopyridinium cations with the Cg···Cg distances of 4.416 (5) Å and 4.102 (5) Å (Cg is the centroide of the pyridine ring) (Fig. 2 and Table 1).

### Experimental

The HCl (5 mL), isonicotinonitrile (20 mmol) and ethanol (50 mL) were added into a 100 mL flask. The mixture was stirred at 333 K for 2 h, and then the precipitate was filtrated out. Colourless crystals suitable for X-ray diffraction were obtained by slow evaporation of the solution.

### Refinement

All the H atoms were situated into the idealized positions and treated as riding with C–H = 0.95 and N—H = 0.90 Å, *U_iso_*(H) = 1.2*U_eq_*(C,N).

### Crystal data

**Table:**

C_6_H_5_N_2_^+^·Cl^−^	*Z* = 2
*M**_r_* = 140.57	*F*(000) = 144
Triclinic, *P*1	*D*_x_ = 1.322 Mg m^−^^3^
Hall symbol: -P 1	Mo *K*α radiation, λ = 0.71073 Å
*a* = 6.6166 (2) Å	Cell parameters from 1231 reflections
*b* = 7.6552 (3) Å	θ = 2.8–27.5°
*c* = 8.3495 (5) Å	µ = 0.45 mm^−^^1^
α = 63.957 (5)°	*T* = 123 K
β = 69.830 (2)°	Block, colorless
γ = 74.367 (4)°	0.10 × 0.05 × 0.05 mm
*V* = 353.16 (3) Å^3^	

### Data collection

**Table:**

Rigaku Mercury2 diffractometer	1231 independent reflections
Radiation source: fine-focus sealed tube	1078 reflections with *I* > 2σ(*I*)
Graphite monochromator	*R*_int_ = 0.042
Detector resolution: 13.6612 pixels mm^-1^	θ_max_ = 25.0°, θ_min_ = 2.8°
CCD profile fitting scans	*h* = −7→7
Absorption correction: multi-scan (*CrystalClear*; Rigaku, 2005)	*k* = −9→9
*T*_min_ = 0.910, *T*_max_ = 1.000	*l* = −9→9
3077 measured reflections	

### Refinement

**Table:**

Refinement on *F*^2^	Primary atom site location: structure-invariant direct methods
Least-squares matrix: full	Secondary atom site location: difference Fourier map
*R*[*F*^2^ > 2σ(*F*^2^)] = 0.086	Hydrogen site location: inferred from neighbouring sites
*wR*(*F*^2^) = 0.234	H-atom parameters constrained
*S* = 1.32	*w* = 1/[σ^2^(*F*_o_^2^) + (0.0002*P*)^2^ + 3.2997*P*] where *P* = (*F*_o_^2^ + 2*F*_c_^2^)/3
1231 reflections	(Δ/σ)_max_ < 0.001
82 parameters	Δρ_max_ = 0.97 e Å^−^^3^
1 restraint	Δρ_min_ = −0.62 e Å^−^^3^

### Special details

**Table:**

Geometry. All esds (except the esd in the dihedral angle between two l.s. planes) are estimated using the full covariance matrix. The cell esds are taken into account individually in the estimation of esds in distances, angles and torsion angles; correlations between esds in cell parameters are only used when they are defined by crystal symmetry. An approximate (isotropic) treatment of cell esds is used for estimating esds involving l.s. planes.
Refinement. Refinement of F^2^ against ALL reflections. The weighted R-factor wR and goodness of fit S are based on F^2^, conventional R-factors R are based on F, with F set to zero for negative F^2^. The threshold expression of F^2^ > 2sigma(F^2^) is used only for calculating R-factors(gt) etc. and is not relevant to the choice of reflections for refinement. R-factors based on F^2^ are statistically about twice as large as those based on F, and R- factors based on ALL data will be even larger.

### Fractional atomic coordinates and isotropic or equivalent isotropic displacement parameters (Å^2^)

**Table:**

	*x*	*y*	*z*	*U*_iso_*/*U*_eq_	
N2	0.8017 (7)	0.1582 (6)	0.6278 (6)	0.0267 (11)	
N1	0.4202 (7)	0.2736 (6)	0.0960 (6)	0.0234 (10)	
H1	0.3576	0.2900	0.0094	0.028*	
C1	0.6318 (8)	0.1817 (7)	0.0856 (7)	0.0238 (12)	
H1A	0.7092	0.1377	−0.0122	0.029*	
C3	0.6118 (8)	0.2221 (7)	0.3646 (6)	0.0192 (11)	
C4	0.3919 (8)	0.3182 (7)	0.3713 (7)	0.0238 (13)	
H4A	0.3103	0.3648	0.4667	0.029*	
C6	0.7156 (8)	0.1908 (7)	0.5071 (7)	0.0258 (13)	
C5	0.3016 (8)	0.3411 (7)	0.2334 (7)	0.0251 (13)	
H5A	0.1565	0.4042	0.2349	0.030*	
C2	0.7328 (8)	0.1531 (7)	0.2198 (7)	0.0222 (12)	
H2A	0.8783	0.0894	0.2146	0.027*	
Cl1	0.1808 (2)	0.31778 (18)	0.82634 (17)	0.0249 (3)	

### Atomic displacement parameters (Å^2^)

**Table:**

	*U*^11^	*U*^22^	*U*^33^	*U*^12^	*U*^13^	*U*^23^
N2	0.031 (2)	0.021 (2)	0.0189 (19)	−0.0006 (18)	−0.0026 (17)	−0.0048 (16)
N1	0.031 (2)	0.0187 (18)	0.0204 (18)	−0.0053 (17)	−0.0098 (16)	−0.0034 (15)
C1	0.028 (2)	0.017 (2)	0.020 (2)	−0.001 (2)	−0.0027 (19)	−0.0056 (18)
C3	0.024 (2)	0.0126 (19)	0.015 (2)	−0.0054 (18)	−0.0029 (18)	−0.0008 (16)
C4	0.023 (2)	0.017 (2)	0.022 (2)	0.0001 (19)	0.0000 (19)	−0.0059 (18)
C6	0.025 (2)	0.023 (2)	0.025 (2)	−0.002 (2)	−0.003 (2)	−0.0097 (19)
C5	0.022 (2)	0.018 (2)	0.029 (2)	−0.0011 (19)	−0.008 (2)	−0.0029 (19)
C2	0.023 (2)	0.016 (2)	0.022 (2)	0.0010 (19)	−0.0046 (18)	−0.0052 (18)
Cl1	0.0244 (6)	0.0273 (6)	0.0235 (5)	0.0001 (5)	−0.0081 (4)	−0.0108 (4)

### Geometric parameters (Å, º)

**Table:**

N2—C6	1.225 (7)	C3—C4	1.437 (7)
N1—C5	1.371 (7)	C3—C6	1.473 (8)
N1—C1	1.379 (6)	C4—C5	1.398 (8)
N1—H1	0.8999	C4—H4A	0.9500
C1—C2	1.404 (8)	C5—H5A	0.9500
C1—H1A	0.9500	C2—H2A	0.9500
C3—C2	1.430 (7)		
			
C5—N1—C1	122.5 (5)	C5—C4—H4A	121.0
C5—N1—H1	118.8	C3—C4—H4A	121.0
C1—N1—H1	118.8	N2—C6—C3	177.9 (5)
N1—C1—C2	119.9 (5)	N1—C5—C4	120.7 (4)
N1—C1—H1A	120.0	N1—C5—H5A	119.6
C2—C1—H1A	120.0	C4—C5—H5A	119.6
C2—C3—C4	120.3 (5)	C1—C2—C3	118.5 (4)
C2—C3—C6	118.9 (4)	C1—C2—H2A	120.7
C4—C3—C6	120.8 (5)	C3—C2—H2A	120.7
C5—C4—C3	118.1 (5)		

### Hydrogen-bond geometry (Å, º)

**Table:**

*D*—H···*A*	*D*—H	H···*A*	*D*···*A*	*D*—H···*A*
N1—H1···Cl1^i^	0.90	2.14	3.033 (5)	174
C4—H4*A*···Cl1	0.95	2.71	3.566 (5)	151
C5—H5*A*···Cl1^ii^	0.95	2.65	3.566 (6)	161

Symmetry codes: (i) *x*, *y*, *z*−1; (ii) −*x*, −*y*+1, −*z*+1.
